# A Cross-Sectional Study on Patient Preferences for Selecting Surgeons for Joint Replacement Surgery in India

**DOI:** 10.7759/cureus.63836

**Published:** 2024-07-04

**Authors:** Mrinal Prakash, Kushal Hippalgaonkar, MV Reddy, Aditya Seth, Tarun Jayakumar, Buddhapuram Pranav Krishna, Praharsha Mulpur, AV Gurava Reddy

**Affiliations:** 1 Orthopedics, KIMS-Sunshine Hospitals, Hyderabad, IND; 2 Orthopedic Surgery, KIMS-Sunshine Hospitals, Hyderabad, IND

**Keywords:** knee arthroplasty, decision making, total knee arthroplasty, surgeon selection, patient preferences

## Abstract

Introduction

This study aims to investigate the complex decision-making process of patients in India when choosing surgeons for joint replacement surgery, with a focus on both clinical and non-clinical factors influencing their preferences.

Methods

This was a cross-sectional observational study conducted at the KIMS-Sunshine Hospitals, Hyderabad, a high-volume tertiary care institute in India, in which patients with end-stage osteoarthritis requiring primary total knee arthroplasty were evaluated using a self-administered questionnaire, which assessed both patient-related and surgeon-related factors in choosing their joint replacement surgeon.

Results

A total of 210 participants were surveyed among whom the majority were females with an average age of 60.2 years with the majority belonging to the upper-middle-class socioeconomic status (48.6%, N=102). Fifty-nine percent preferred surgeons with over 20 years of experience, and 63.8% were willing to travel out-of-state for recognized expertise. Family recommendations (33.8%) and surgeon reputation (24.3%) were primary factors in surgeon selection. A vast majority (73.3%) preferred surgeons who were skilled in robotic surgery and had foreign training (32.9%). However, the majority (67.6%) did not express any gender preference. The survey highlighted a broad range of informational sources affecting decisions, including financial consideration (63.8%), personal referrals, and online platforms (17.1%). Preferences were also shaped by hospital reputation and insurance options (10.5%), illustrating a nuanced interplay of quality, cost, and personal connections in the selection process.

Conclusion

The findings of this survey illuminate the intricate and diverse preferences exhibited by patients when selecting a surgeon for joint replacement surgery. A significant rise in patient expectations is evident, underscoring a demand for more personalized, contemporary, and high-quality healthcare services. Importantly, geographical proximity appears to be a diminishing concern in their decision-making process. This trend presents an opportunity for centers of excellence to extend their influence and attract patients on both a regional and national level.

## Introduction

Over the past several decades, the field of orthopedics, particularly in joint replacement surgery, has experienced significant innovations. Joint replacement surgery has emerged as a highly effective intervention for alleviating pain and enhancing mobility in individuals suffering from severe joint arthritis [1]. In India, there has been a marked increase in the prevalence of these procedures, driven by improved healthcare infrastructure, advancements in literacy, widespread internet access, and proactive governmental initiatives aimed at providing affordable healthcare [2]. Moreover, the imposition of price-capping regulations has significantly increased the accessibility of these surgeries to a wider segment of the population. This regulatory change has stimulated growth in the surgical implant market for Indian and multinational companies, including local manufacturing [2].

The dynamics of healthcare decision-making are rapidly evolving, mainly characterized by a growing recognition of patients' active roles in selecting their healthcare providers [3]. In the Indian scenario, where the healthcare sector is witnessing profound transformations, patients are actively participating in the selection of their joint replacement surgeons, recognizing the profound impact this choice has on their well-being [4]. As healthcare consumers, individuals are no longer passive recipients but proactive participants in a complex decision-making process [4].

A study conducted by Losina et al., involving 932 Medicare beneficiaries who underwent total knee replacement surgery in 2000 highlighted that the predominant factor influencing the choice of hospital was the surgeon's reputation, with 68% of patients citing it as their primary consideration [5]. Similarly, Schwartz et al. reported that the reputation of the surgeon was the key determinant in selecting a healthcare facility for major surgical interventions, with 80% of the 510 patients interviewed emphasizing its importance in their decision-making process [6].

However, the process of selecting a joint replacement surgeon in India involves multiple dimensions. Patients consider clinical factors, such as the surgeon's expertise and success rate, as well as recommendations from their general physicians [7]. Socioeconomic factors are also significant, as the costs associated with the surgery and post-operative care are crucial, particularly given that many Indian patients lack health insurance [8]. Cultural factors are equally pertinent, with patients often preferring surgeons who respect and understand their cultural values. Additionally, access to specialized healthcare facilities can greatly influence patient decisions regarding joint replacement surgery [8].

The decision to choose a surgeon, especially in elective specialty surgeries, is often challenged by limited available information [8]. Patients frequently rely on the Internet as a crucial resource for information about their health and potential healthcare providers [9]. The impact of internet usage and social media on healthcare choices is profound, facilitating the exchange of information, experiences, and perspectives among patients [10].

Recent market surveys indicate a consistent annual increase in joint replacement surgeries in India and understanding the decision-making process of patients is crucial for healthcare providers [11]. This study aims to explore the comprehensive range of factors influencing the choice of joint replacement surgeons in India, incorporating both clinical and non-clinical criteria. By examining these factors, we hope to provide insightful contributions to the Indian healthcare landscape, enabling providers to better meet the evolving preferences and needs of patients.

## Materials and methods

This was a cross-sectional observational study conducted at a single high-volume tertiary care institute specializing in joint replacement surgery, the KIMS-Sunshine Hospitals, Hyderabad, from October 1, 2023, to October 31, 2023. The study was conducted in compliance with the Declaration of Helsinki [12], received an exemption from the Institutional Ethics Committee, KIMS-Sunshine Hospitals, and is reported to adhere to STROBE (STrengthening the Reporting of OBservational studies in Epidemiology) guidelines [13].

Patients with end-stage osteoarthritis of the knee who were counseled for primary total knee arthroplasty were included in the study after taking written informed consent. Exclusion criteria consisted of patients requiring revision surgeries, prior history of infection, prior surgeries to the knee, mental health disorders, inability to comprehend the language, and patients refusing to participate in the study. The anonymity of participants was strictly maintained throughout the process of data collection and analysis.

Study questionnaire

Patient demographic details, living area (urban/rural), and socioeconomic status were collected and evaluated using the Modified Kuppuswamy Socioeconomic Scale (SES), 2023 [14]. Data was gathered through a self-administered questionnaire aimed at evaluating patient’s demographic data and their preferences for selecting an arthroplasty surgeon. An arthroplasty research fellow was assigned to clarify doubts and help patients in filling the questionnaire. The questionnaire was divided into two parts, consisting of a total of 17 questions. The first part of the questionnaire comprised questions about patient characteristics, while the second part focused on surgeon characteristics.

The first part included eight distinct questions exploring the patient-related factors that influence decision-making. Participants were asked how they initially became aware of the option of joint replacement surgery, shedding light on the sources of information available to them. Patients were asked about their knowledge regarding major orthopedic surgeon degrees, crucial factors that exerted influence during the surgeon selection process, factors that convinced them to undergo the procedure, and obstacles that may have previously hindered them from deciding to undergo surgery. Geographical factors were evaluated by asking the patients how distance from their residence influenced the choice of healthcare facility in the same city and if they were willing to travel to a different city to seek out a renowned surgeon. The role of financial factors in surgeon selection was evaluated as it forms an integral part of the decision-making process in India.

The next set of nine questions was meticulously designed to uncover the surgeon-related factors influencing patients' choices. It included surgeon's experience, educational qualification, international training, academic contributions, specialized training in robotics, and advanced qualifications in arthroplasty. The role of patients' interactions with surgeons was evaluated. Patients were asked to express their preferences for orthopedic surgeons based on gender, thus recognizing the potential influence of this demographic factor on surgeon selection. These questions focusing on surgeon characteristics provided an in-depth understanding of the multifaceted considerations guiding patients in choosing their joint replacement surgeon.

Validation of the questionnaire involved re-asking the same questions to the first 20 patients after a 10-day interval, and comparing their new answers to their previous responses.

Statistical analysis

Microsoft Excel (Microsoft Corp., Redmond, WA) and IBM SPSS Statistics for Windows, Version 24 (IBM Corp., Armonk, NY) were employed for statistical analysis. Open-ended questions primarily focused on baseline information such as age, location, and education details. Descriptive statistics were used to analyze continuous variables which were represented using mean, standard deviation, and range; while categorical variables were depicted through frequencies and percentages.

## Results

A total of 210 participants were surveyed in this cross-sectional study. Thirteen (6.2%) participants did not fill in their sociodemographic credentials due to privacy issues. Participants had a mean age of 60.2 years (range: 40-85 years). The majority of the patient population surveyed were females (56.2%, n=118) belonging to the upper-middle class background (48.6%, n=102) according to the Modified Kuppuswamy SES as shown in Table 1. 

**Table 1:** Patient demographics Modified Kuppuswamy Socioeconomic Scale, 2023 [14]

Parameter	Value
Age (Mean, SD)	60.4 (9.8)
Gender, female (N, %)	119 (56.6)
Living area (N, %)	
- Urban	135 (64.3)
- Rural	75 (35.7)
Modified Kuppuswamy Socioeconomic Scale, 2023 (N, %)	
- Upper (I)	30 (14.3)
- Upper middle (II)	102 (48.6)
- Lower middle (III)	36 (17.2)
- Upper lower (IV)	20 (9.5)
- Lower (V)	22 (10.4)

Patient-related questions

Awareness of joint replacement surgery among the study participants was primarily through personal experiences shared by operated patients or their relatives, which accounted for 42.9% (n=90) of responses. Orthopedic surgeon referrals were the next significant source of awareness, cited by 29.5% (n=62) of participants, highlighting the trust placed in professional advice. Online sources, including health websites and forums, were instrumental for 21.4% (n=45) of the survey participants, reflecting the increasing role of digital media in healthcare decision-making. Traditional media sources such as newspapers, television news, and radio were less influential, with only 6.2% (n=13) relying on them.

A considerable majority of participants, 76.7% (n=161), reported that they actively inquired about a surgeon’s qualifications before scheduling a consultation. This high level of engagement indicates a significant concern for the surgeon's professional credentials and expertise, underscoring the importance of transparency in the surgeon's educational and training background. The survey findings are summarized in Table 2.

**Table 2:** Patient-related questions

	Question	Options	Result N (%)
1	How did you come to know about joint replacement surgery?	Orthopedic surgeon	62 (29.5)
		Online	45 (21.4)
		Operated patients or their relatives	90 (42.9)
		Newspaper, TV news, radio	13 (6.2)
2	Do you enquire about the surgeon’s degree before you consult?	Yes	161 (76.7)
		No	49 (23.3)
3	What was the single most important factor that influenced surgeon selection?	Recommendation by family member	71 (33.8)
		Recommendation by family doctor	30 (14.3)
		Surgeon’s name and fame	51 (24.3)
		Hospital’s reputation and insurance services available	22 (10.5)
		Patient testimonials, online reviews	36 (17.1)
4	Would you travel to a different state in the country to seek a renowned surgeon?	Yes	134 (63.8)
		No	76 (36.2)
5	Do you consider the cost of surgery when selecting a surgeon?	Yes	134 (63.8)
		No, I would go for the best surgeon even if I have to borrow money or take a loan	76 (36.2)
6	Which factor influenced you most in the decision to undergo joint replacement surgery?	Interaction with the doctor	102 (48.6)
		Prospect of avoiding pain medication	30 (14.3)
		X‐rays revealing deformity and reduced joint space between the bones	40 (19.0)
		Hearing positive experiences of other patients	38 (18.1)
7	If choosing within your city, how important is the distance of the hospital from your home?	Very important	119 (56.7)
		Not important, I would go for the best option	91 (43.3)
8	What’s the biggest obstacle that has prevented you from deciding on surgery in the past?	Lack of knowledge of the severity of arthritis	48 (22.8)
		Lack of knowledge of the procedure	36 (17.1)
		Financial issues	21 (10)
		Fear of surgery and post-operative pain	62 (29.5)
		Bad experiences of other patients	25 (12)
		No social and moral support	3 (1.4)
		Fear of age-related factors while undergoing major surgery	15 (7.2)

Surgeon-related questions

The selection of a surgeon was heavily influenced by recommendations from family members, with 33.8% (n=71) of participants stating this was their primary consideration. This suggests that personal trust and experiences relayed through close social connections play a critical role in the decision-making process. The reputation of the surgeon, as a standalone factor, influenced 24.3% (n=51) of the survey population, indicating that perceived competence and recognition in the field are highly valued.

Recommendations from family doctors were significant for 14.3% (n=30) of participants, highlighting the influence of primary care providers in specialist selection. Additionally, patient testimonials and online reviews collectively impacted 17.1% (n=36) of participants, demonstrating the growing importance of peer opinions and digital reputation in healthcare choices. The reputation of the hospital and the availability of comprehensive insurance options were considered important by 10.5% (n=22), reflecting considerations of overall healthcare quality and financial planning.

A strong majority of the participants, 63.8% (n=134), were willing to travel to a different state to access a renowned surgeon, suggesting that the reputation and expertise of the surgeon are considered more crucial than geographic convenience. Similarly, cost considerations played a major role, with the same percentage of participants (63.8%, n=134) factoring it into their decision, indicating a significant concern over the affordability of surgery. This willingness to travel and consider cost highlights a trend toward prioritizing quality and expertise over proximity and potentially lower costs. The survey findings are summarized in Table 3.

**Table 3:** Questions related to the surgeon’s education, training, and gender MS: Master of Surgery, DNB: Diplomate of National Board, MCh: Master of Chirurgiae

	Questions	Options	Result N (%)
1	How much importance do you give to the number of years of experience of the surgeon?	Should be more than 20 years	124 (59)
		Should be more than 10 years	52 (24.8)
		Irrelevant if source of referral is trusted	34 (16.2)
2	Surgeon with which academic degree would you prefer?	MS orthopedics	93 (44.3)
		DNB orthopedics	16 (7.6)
		Not aware about these degrees	71 (33.8)
		Aware but no such preference	30 (14.3)
3	Would you prefer an orthopedic surgeon who specializes only in joint replacement surgeries over a general orthopedic surgeon?	Yes	138 (65.7)
		No	72 (34.3)
4	Would you prefer a surgeon trained in a foreign country?	Yes	69 (32.9)
		No	141 (67.1)
5	How would you choose a surgeon based on the interaction you had with the surgeon?	A famous surgeon, irrespective of the duration of interaction with him	122 (58.1)
		A young surgeon who gives more time and takes active participation in decision-making	88 (41.9)
6	Would you prefer a surgeon who has done MCh/Fellowship in joint replacement surgery?	Yes	157 (74.8)
		No	53 (25.2)
7	Does it matter if the surgeon has published articles and research papers on joint replacement surgery?	Yes	93 (44.3)
		No	117 (55.7)
8	Would you prefer a surgeon who does robotic joint replacement surgery?	Yes	154 (73.3)
		No	56 (26.7)
9	If given an option, which orthopedic surgeon would you prefer?	Male orthopedic surgeon	43 (20.5)
		Female orthopedic surgeon	25 (11.9)
		No such preference	142 (67.6)

## Discussion

The findings from this study offer insightful perspectives on patient preferences regarding the selection of surgeons for joint replacement surgeries. These insights not only reflect the changing dynamics of patient engagement in healthcare decisions but also underscore the importance of various factors beyond the surgeon's technical expertise. These preferences span across a broad spectrum of considerations including the surgeon's experience, training, specialization, interaction level, advanced qualifications, published research, approach to surgery (specifically robotic surgery), and even the surgeon's gender. Studies done in surgical patient populations revealed surgeon reputation, training, certification, and technical expertise to be the important factors contributing to surgeon selection by patients [6,15-20].

The emphasis on a surgeon’s experience, with a significant majority of patients preferring surgeons with over 20 years of experience, reflects a traditional view where longevity in practice is equated with skill and reliability [21]. The survey responses indicate a strong preference for experienced surgeons with advanced qualifications, reflecting a belief that more experience and specialized training correlate with better outcomes [21]. This is contrasted by a considerable fraction of the population who value the source of referral over the years of experience, suggesting a trust-based approach in surgeon selection. When we explored patient awareness regarding the qualifications of orthopedic surgeons in India, we discovered that a significant proportion of the patient population, 71 out of 210 individuals (33.8%), lacked knowledge about the fundamental degrees required for practicing orthopedics in India. Interestingly, a smaller proportion of the respondents, 49 patients (23.3%), reported that they did not inquire about the surgeon's qualifications prior to their consultation. This discrepancy suggests that, despite a lack of familiarity with the specific educational credentials, patients often form perceptions of a surgeon's expertise based on other factors, such as the surgeon's city of practice, alma mater, or international training experiences [15]. This observation underscores the importance of patient education on professional qualifications and highlights the diverse criteria patients may use to assess healthcare providers. The preference for surgeons with an MS (Master of Surgery) in Orthopedics over other qualifications suggests a perceived hierarchy in educational credentials, which may influence patient confidence in surgical outcomes [6]. Past studies indicated board certification and specialized training as the factors with the highest patient ratings in surgeon selection [6,15]. Approximately one-third of the respondents (32.9%, or 69 people) favored surgeons with training from foreign countries, while the greater portion (67.1%, or 141 people) indicated they had no specific preference for the geographical location of the surgeon's training. Patient’s preference for surgeons with advanced qualifications like an MCh (Master of Chirurgiae) or Fellowship in Joint Replacement Surgery highlights the importance of perceived competence through additional training. The interest in surgeons who perform robotic surgery demonstrates a patient preference for practitioners who are perceived as being at the forefront of medical technology and knowledge. However, the mixed importance placed on published research by surgeons suggests that while academic contributions to the field are important to some, others may prioritize practical experience or outcomes more heavily. In our study, a significant portion of respondents with no preference regarding the surgeon's gender suggests a shift towards valuing professional qualifications and personal interactions over traditional gender biases [22-24]. Among the 25 patients who expressed a preference for a female surgeon, 24 were female. Furthermore, of these 25 patients, 13 were aged 60 years or older.

In our study, the primary influencer in choosing a surgeon was a recommendation from a family member, followed by the surgeon's reputation. Other factors taken into account included referrals from family doctors, feedback from other patients, and online reviews, as well as the reputation of the hospital and its insurance provisions. Patients placed greater importance on recommendations from former patients, family members, friends, or other physicians over the recognition or advertisements from physicians. Likewise, previous surveys have shown that patients heavily depend on recommendations from family, friends, and other physicians when choosing specialists [25-27]. Conversely, advertisements on radio, the Internet, and television have been linked to lower patient reliance, likely because of the abundance of commercials promoting the "most advanced medical center" or the "best physician" across various platforms [15]. In our study, however, the substantial number of patients verifying surgeon credentials independently signals a shift towards more informed and proactive patient behavior. This dual reliance on both personal and formal sources of information suggests that surgeons and healthcare institutions must maintain a robust and positive presence both in their direct communities and online.

Interactions with the doctor being the most influential factor in deciding to undergo surgery reinforces the importance of the surgeon-patient relationship. It suggests that how surgeons communicate, empathize, and engage with patients can significantly impact patient decisions [20,28,29]. This finding resonates closely with the work of Bozic et al., where among 251 patients undergoing elective total joint arthroplasty, the surgeon's demeanor and the quality of information provided were identified as the most crucial factors [30,31]. Moreover, the primary deterrents to surgery were fear of the procedure and postoperative pain, alongside financial concern; all of which point to areas where better patient education and support systems could alleviate apprehensions. It indicates that the way surgeons communicate, show empathy, and engage with patients can significantly sway patient decisions [4,19].

Approximately two-thirds of the patients (63.8%, or 134 individuals) expressed a willingness to travel to another state for surgery if it meant receiving treatment from a renowned surgeon. Conversely, 36.2% (76 individuals) preferred to avoid traveling. Similar, to our results, hospital reputation, location, parking availability, and home-to-clinic distance have been the least important factors considered by patients [6,32,33]. Regarding, cost considerations, 63.8% (134 individuals) of patients cited cost as a determining factor when selecting a surgeon, while the remaining 36.2% (76 individuals) placed greater importance on quality, even indicating a readiness to borrow money or seek a loan if required. The divided opinions on cost considerations reveal a healthcare consumer mindset where quality often supersedes financial constraints [34].

The demographic makeup of the participants, showing a larger proportion of females, could indicate broader societal patterns or particular inclinations within the population toward being aware of joint health. The broad age range underscores the significance of joint replacement surgery throughout a substantial portion of adult life, highlighting the need for a varied approach to patient communication and education.

The findings from this study underscore several key implications for orthopedic practice and healthcare policy. Firstly, there is a crucial need for enhanced patient education initiatives to address and dispel common myths and anxieties associated with joint replacement surgery and the subsequent healing process. Secondly, the interaction between patients and surgeons plays a pivotal role in the decision-making process; therefore, surgeons and healthcare providers must focus on building trust through effective communication strategies. Thirdly, transparency regarding the financial costs, expected outcomes, and associated surgery expenses is essential for patients to make informed decisions. Lastly, embracing and implementing cutting-edge surgical methods and showcasing advanced credentials can attract individuals seeking state-of-the-art care, thereby positioning the practice at the forefront of modern healthcare.

This study, while comprehensive in its scope and execution, has several limitations. Firstly, the study conducted at a single high-volume tertiary care institute limits the generalizability of the findings to other settings, particularly smaller hospitals or rural healthcare facilities in India. Additionally, excluding those unable to comprehend the questionnaire further narrows the study population, potentially omitting important perspectives and the focus on a specific geographic region also limits the applicability of the results to a more diverse national population. Future studies should aim to include a more diverse patient population across multiple centers and employ longitudinal designs better to understand the dynamics of patient decision-making in surgeon selection.

## Conclusions

The findings of this survey illuminate the intricate and diverse preferences exhibited by patients when selecting a surgeon for joint replacement procedures. The data highlight a significant rise in patient expectations, underscoring a demand for more personalized, contemporary, and high-quality healthcare services. Importantly, geographical proximity appears to be a diminishing concern in their decision-making process. This trend presents an opportunity for centres of excellence to extend their influence and attract patients on both a regional and national level.
